# Synthesis, PASS-Predication and in Vitro Antimicrobial Activity of Benzyl 4-*O*-benzoyl-α-l-rhamnopyranoside Derivatives

**DOI:** 10.3390/ijms17091412

**Published:** 2016-08-27

**Authors:** Mohammed Mahbubul Matin, Amit R. Nath, Omar Saad, Mohammad M. H. Bhuiyan, Farkaad A. Kadir, Sharifah Bee Abd Hamid, Abeer A. Alhadi, Md. Eaqub Ali, Wageeh A. Yehye

**Affiliations:** 1 Organic Research Laboratory, Department of Chemistry, University of Chittagong, Chittagong 4331, Bangladesh; mosharefchem@cu.ac.bd; 2 Nanotechnology & Catalysis Research Centre (NANOCAT), University of Malaya, Block 3A, Institute of Postgraduate Studies Building, Kuala Lumpur 50603, Malaysia; amit_cu.chem@yahoo.com (A.R.N.); sharifahbee@um.edu.my (S.B.A.H.); eaqubali@um.edu.my (M.E.A.); 3 Department of Pharmacy, Faculty of Medicine, University of Malaya, Kuala Lumpur 50603, Malaysia; omar79@siswa.um.edu.my; 4 Division of Human Biology, School of Medicine, International Medical University, Kuala Lumpur 57000, Malaysia; faalhadi@yahoo.com; 5 Department of Chemistry, Faculty of Science, University of Malaya, Kuala Lumpur 50603, Malaysia; aaalhamadani@yahoo.com

**Keywords:** l-rhamnose, benzyl α-l-rhamnopyranoside, benzoylation, prediction of activity spectra for substances (PASS), structure activity relationship, antimicrobial agents

## Abstract

Benzyl α-l-rhamnopyranoside **4**, obtained by both conventional and microwave assisted glycosidation techniques, was subjected to 2,3-*O*-isopropylidene protection to yield compound **5** which on benzoylation and subsequent deprotection of isopropylidene group gave the desired 4-*O*-benzoylrhamnopyranoside **7** in reasonable yield. Di-*O*-acetyl derivative of benzoate **7** was prepared to get newer rhamnopyranoside. The structure activity relationship (SAR) of the designed compounds was performed along with the prediction of activity spectra for substances (PASS) training set. Experimental studies based on antimicrobial activities verified the predictions obtained by the PASS software. Protected rhamnopyranosides **5** and **6** exhibited slight distortion from regular ^1^C_4_ conformation, probably due to the fusion of pyranose and isopropylidene ring. Synthesized rhamnopyranosides **4**–**8** were employed as test chemicals for in vitro antimicrobial evaluation against eight human pathogenic bacteria and two fungi. Antimicrobial and SAR study showed that the rhamnopyranosides were prone against fungal organisms as compared to that of the bacterial pathogens. Interestingly, PASS prediction of the rhamnopyranoside derivatives **4**–**8** were 0.49 < *P*_a_ < 0.60 (where *P*_a_ is probability ‘to be active’) as antibacterial and 0.65 < *P*_a_ < 0.73 as antifungal activities, which showed significant agreement with experimental data, suggesting rhamnopyranoside derivatives **4**–**8** were more active against pathogenic fungi as compared to human pathogenic bacteria thus, there is a more than 50% chance that the rhamnopyranoside derivative structures **4**–**8** have not been reported with antimicrobial activity, making it a possible valuable lead compound.

## 1. Introduction

**Figure 1:**
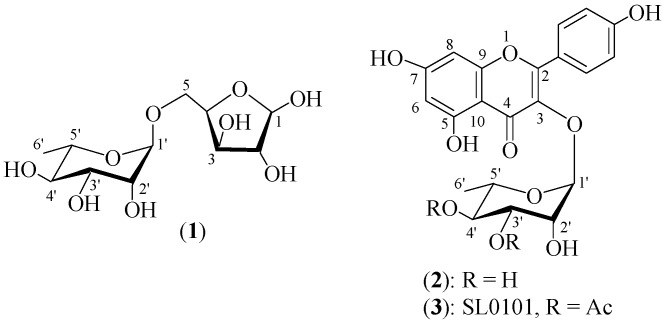
Naturally occurring important rhamnopyranosides (**1**), (**2**) and (**3**).

l-Rhamnose is widely distributed in nature and is found as a constituent of plant glycosides, gums and also in bacterial polysaccharides [1]. The presence l-rhamnose, as the aglycone moiety, in various oligosaccharides was found to be essential for identification of immunodominant groups in polysaccharides which have antigenic activity [2]. For example, 5-*O*-α-l-rhamnopyranosyl-β-l-arabinofuranose **1** in Figure 1 was found as the sugar component of sitosterol glycoside, which exhibited rhamnosidase specificity in *Aspergillus niger* [3]. Kaempferol-3-*O*-(3′,4′-di-*O*-acetyl-α-L-rhamnopyranoside) **2** was isolated from nature. Its diacetyl derivative, also called SL0101 **3**, is a highly specific protein kinase (RSK) inhibitor [4] and was isolated from *Forsteronia refracta*. Diacetyl compound **3** was found to have 12 times more in vitro RSK inhibitor than that of its non-acetyl analogue **2**. It was concluded that acylation of the rhamnose moiety in these natural products is essential for high affinity binding and selectivity as well as for the development of anticancer agents (RSK inhibitors) [4].

The emergence of multiple antibiotic resistant pathogenic bacteria causes major threat to human health worldwide. In this context, search for new antibacterial agents with novel mode of action represents a major target in chemotherapy [5]. Acylated sugars (sugar esters) attracted considerable research interest in recent years because of their diverse and remarkable biological activities such as anticancer agents [6], insecticides [7], antibacterial agents [8,9,10] and antifungal [11] activities. These esters have also been widely used as cosmetic and in pharmaceutical industries for many years as they are considered biocompatible, biodegradable, and nontoxic [12,13]. The sugar moieties of acyl esters can increase drug water solubility, decrease toxicity and contribute to the bioactivity of the natural products. In addition, incorporation of acyl nuclei with sugar residue especially aromatic nuclei enhances the biological profile many times than that of the parent sugar [14].

Protected monosaccharide derivatives are used as intermediate for many biologically important natural products [15,16,17]. Partially protected rhamnose monomers especially acylated rhamnose are very useful building blocks for further transformation to these compounds. Although, selective protection of monosaccharide molecules is a prominent challenge as they contain several hydroxyl groups of similar reactivity. Small differences in reactivity cannot be utilized in this respect. However, desired protection can be achieved in one or few steps employing complex reaction sequences [18]. Such as, organotin reagents, tributyltin oxide or dibutyltin oxide [19,20] are often used to accomplish regioselective protection, including acylation [21,22] of monosaccharide derivatives. In case of rhamnopyranoside, the regioselectivity is difficult to be controlled due to the similarity of the secondary 2-, 3- and 4-trihydroxyls [20,23,24,25]. When rhamnopyranosides were subjected for selective acylation employing dibutyltin oxide mediated method, it furnished corresponding 3-*O*-acyl derivatives only [20,23,24,25]. Even direct unimolecular acylation also provided a mixture of 2- and/or 3-*O*-acylates. Considering the immense importance of 4-*O*-acyl esters [4] of this l-rhamnose, we have designed the synthesis of some 4-*O*-benzoyl derivatives of benzyl α-l-rhamnopyranoside (**4)**, using protection deprotection technique, keeping view that these compounds will contain aromatic moiety in its molecular framework, which are expected to be more toxic against pathogenic microorganisms. The prediction of activity spectra for substances (PASS) predicted more than 300 pharmacological effects, biological and biochemical mechanisms based on the structural formula of the substance [26,27,28]. This was efficiently used in this study to support new antimicrobial actions for the experimentally verified compounds.

## 2. Results and Discussion

We mainly describe the synthesis of benzyl 4-*O*-benzoyl-α-L-rhamnopyranoside **7** employing protection deprotection technique. For comparison of antimicrobial study, 2,3-di-*O*-acetate **8** was also prepared.

### 2.1. Synthesis of Benzyl 4-O-benzoyl-α-L-rhamnopyranoside **7**

**Scheme 1:**
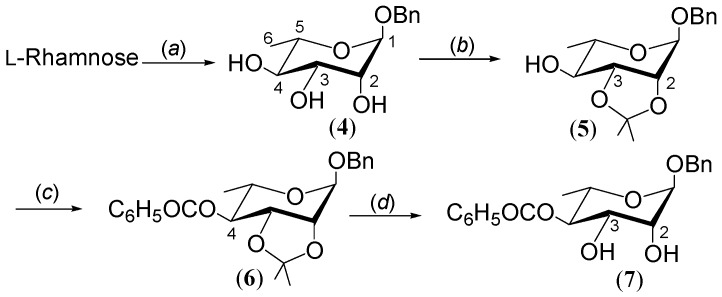
Reagents and conditions: (**a**) reference [20], 82%, or microwave irradiation (MWI), 90 s, 96%; (**b**) 2,2-dimethoxypropane (2,2-DMP), *p*-toluenesulfonic acid (*p*-TSA), room temperature (rt), 2 h, 93%; (**c**) C_6_H_5_COCl, pyridine, 4-dimethylaminopyridine (DMAP), 0 °C–rt, 12 h, 95%; and (**d**) AcOH, 40 °C, 18 h, 82%. (**4**): Benzyl α-l-rhamnopyranoside; (**5**): Benzyl 2,3-*O*-isopropylidene-α-l-rhamnopyranoside; (**6**): Benzyl 4-*O*-benzoyl-2,3-*O*-isopropylidene-α-l-rhamnopyranoside; and (**7**): Benzyl 4-*O*-benzoyl-α-l-rhamnopyranoside.

As discussed earlier that the acylation of benzyl α-l-rhamnopyranoside **4** employing dibutyltin oxide method furnished the 3-*O*-acyl derivatives only [23,24,25,29]. For 4-*O*-benzoylation, we initially prepared benzyl α-l-rhamnopyranoside **4** from l-rhamnose using reported procedure [20] (Scheme 1) in good yield. Fourier transform infrared spectroscopy (FT-IR) spectrum of this compound **4** exhibited characteristic broad stretching band at 3480–3310 cm^−1^ similar to the hydroxyl group(s).

In addition to this conventional glycosidation, we applied microwave irradiation (MWI) to a mixture of powdered L-rhamnose with little excess dry benzyl alcohol (in a porcelain dish) and Amberlite ionomer resin (IR) 120 (H^+^) ion exchange resin at 160 watts for 90 s in a domestic microwave oven. After chromatographic purification, benzyl rhamnopyranoside **4** was isolated almost in quantitative (96%) yield. Thus, MWI method (only 90 s) was found to be suitable over conventional method (20 h). Having rhamnopyranoside **4** in hand, we attempted protection of its C-2 and C-3 positions as isopropylidene group. Thus, reaction of **4** with 2,2-dimethoxypropane (DMP, excess) in the presence of catalytic amount of *p*-toluenesulfonic acid (*p*-TSA) gave an oil. In its FT-IR spectrum, a broad stretching band at 3450–3300 was assigned for hydroxyl group(s) and a sharp band at 1381 cm^−1^ arise due to incorporation of isopropylidene group. This was further confirmed by analyzing its ^1^H nuclear magnetic resonance (NMR) spectrum, wherein two three-proton singlets appeared at δ 1.33 and 1.32, corresponding to two methyl groups of one isopropylidene group. Thus, one acetonide group must be incorporated in the product molecule. Complete analysis of its FT-IR and proton NMR spectra led us to assign the compound as benzyl 2,3-*O*-isopropylidene-α-l-rhamnopyranoside **5**. Reasonably isopropylidene ring was formed in the *cis*-vicinal diol (C-2 and C-3) positions of rhamnopyranoside **4** and similar observation was reported by Lazar et al. [30].

In the subsequent step, compound **5** having C-4 free hydroxyl group was reacted with benzoyl chloride (1.1 equivalent) in dry pyridine to furnish a white solid, melting points (mp) 115–117 °C (Scheme 1). We observed complete disappearance of hydroxyl stretchings in the FT-IR spectrum of this solid and appearance of a new stretching band at 1681 cm^−1^ which indicated the presence of a carbonyl group. In its ^1^H NMR spectrum, a total of ten aromatic protons resonated at δ 8.04–8.226 (4H, m), 7.58–7.73 (2H, m) and 7.35–7.52 (4H, m). The presence of addition five protons as compared to **5** clearly indicated the incorporation of one benzoyloxy group in this molecule. The attachment of the benzoyloxy group at C-4 position of this molecule was confirmed by observing the fact that H-4 resonated downfield at ~δ 5.18 ppm as compared to the precursor compound **5** (δ 4.42–4.48 ppm). So, the structure of this compound was unambiguously established as benzyl 2,3-*O*-isopropylidene-4-*O*-benzoyl-α-l-rhamnopyranoside (**6**).

Finally, we attempted for the removal of isopropylidene protecting group from compound **6**. Thus, 4-*O*-benzoate **6** was stirred with glacial acetic acid at 40 °C for 18 h to furnish a semi-solid (Scheme 1). The appearance of a new broad band in its FT-IR spectrum at 3600–3300 cm^−1^ similar to hydroxyl stretching and disappearance of isopropylidene stretching (1375 cm^−1^) clearly indicated that isopropylidene group was removed from the molecule. In its ^1^H NMR spectrum, signals corresponding to methyl protons of isopropylidene group were disappeared instead a new two-proton broad singlet at δ 1.67–2.19 corresponding to two hydroxyl groups was observed. These observations further confirmed the removal of isopropylidene group from this molecule. Upon complete analysis of its FT-IR and proton NMR spectra, the structure of the compound was established as benzyl 4-*O*-benzoyl-α-L-rhamnopyranoside (**7**).

### 2.2. Synthesis of 2,3-di-O-Acetyl Derivative of 4-O-Benzoate **7**

**Scheme 2:**
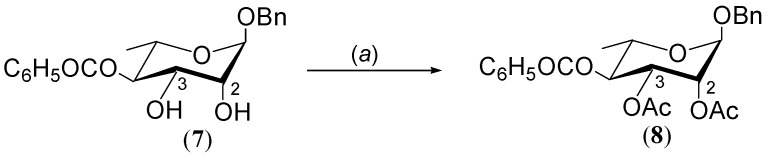
Reagents and conditions: (**a**) Ac_2_O, pyridine, DMAP, 0 °C–rt, 12 h, 92%. (**8**): Benzyl 2,3-di-*O*-acetyl-4-*O*-benzoyl-α-L-rhamnopyranoside.

After successful synthesis of 4-*O*-benzoate **7**, we planned to confirm its structure further and prepare newer rhamnopyranoside derivative(s) of biological significance. Thus, as presented in Scheme 2, acetylation of diol **7** with little excess acetic anhydride in anhydrous pyridine gave a syrup in good yield (92%). FT-IR spectrum of this syrup gave signals at 1751, 1732 and 1716 cm^−1^ (CO) and showed absence of signals corresponding to hydroxyl stretching indicating acetylation of the product molecule. Again, in its ^1^H NMR spectrum, two three-proton singlets at δ 2.16 and 1.88 corresponding to two acetyl-methyl groups further indicated the incorporation of two acetyloxy groups in the same molecule. Additionally, H-2 (δ 5.33) and H-3 (δ 5.54) protons of this compound shifted considerably downfield as compared to its precursor compound **7** (~δ 4.07–4.09). These observations clearly proved that the two acetyloxy groups were introduced at C-2 and C-3 positions. So, structure of the compound was established as benzyl 2,3-di-*O*-acetyl-4-*O*-benzoyl-α-l-rhamnopyranoside (**8**).

### 2.3. Conformational Study: Distortion of Rhamnopyranosides **5** and **6**

Methyl α-l-rhamnopyranoside and benzyl α-l-rhamnopyranoside **4** are well-known to exist in regular ^1^C_4_ conformation [31,32]. Rhamnopyranosides with regular ^1^C_4_ conformation generally exhibit coupling constant between *cis*-axial equatorial H-2 and H-3 (*J*_2,3_) ~3.0 Hz, between *trans*-diaxial H-3 and H-4 (*J*_3,4_) ~10 Hz and between *trans*-diaxial H-4 and H-5 (*J*_4,5_) ~10 Hz. From the coupling constant (*J*) values calculated from ^1^H NMR spectral data (400 MHz, CDCl_3_), as shown in Table 1, almost similar coupling constants, i.e., ^1^C_4_ conformation, was observed for rhamnopyranosides **7**–**8**. However, in case of its protected rhamnopyranosides **5** and **6**, the presence of isopropylidene functionality at C-2 and C-3 position and/or acyl group(s) increases the bulk in these molecules. In case of compound **5**, *J*_2,3_ was found higher (5.8 Hz) and *J*_3,4_ was found lower (6.9 Hz) than that of the normal *J* value. This deviation of *J* value(s) indicated that conformation of compound **5** must be distorted from regular ^1^C_4_ conformation. We believe that this distortion occurred due to the fusion of bulky five-membered isopropylidene group with six-membered pyranose sugar ring. Similarly, compound **6** showed *J*_4,5_ = 6.9 Hz, which is significantly lower than that of the normal value. Hence, compound **6** must be distorted from regular ^1^C_4_ conformation.

### 2.4. Computational Evaluation of Antimicrobial Activities

PASS programme is designed to anticipate more than 4000 forms of biological activity including drug and non-drug actions and can be employed to identify the most probable targets with 90% accuracy [26,33,34,35]. This PASS prediction is well established by the successful application and confirmation in the pharmacological research experiments [36,37,38,39]. PASS result is designated as *P*_a_ (probability for active compound) and *P*_i_ (probability for inactive compound). For example, *P*_a_ > 0.7 indicates the possibility of discovering the highly active compound in the experiment while 0.5 < *P*_a_ < 0.7 expresses the possibility of getting less active one and dictates the different structure of the compound from the PASS training set. On the other hand, *P*_a_ < 0.5 means the possibility of finding the activity is very much less in the experiment. Even if greater than 50% possibility structure reported with no particular biological activity in context is verified, it might be a main compound. However, PASS predictions can only elucidate the intrinsic activity of a compound through experimentation. In this study, PASS was used to explore the antimicrobial activities of 4-*O*-benzoyl derivatives of benzyl α-l-rhamnopyranoside **5**. Publications, patents, databases, and private communications were used as the source data of the PASS training set for the SAR analysis to predict the potential antimicrobial activities of the synthesized compounds. Therefore, antimicrobial activities of the synthesized compounds can be categorized either antibacterial or antifungal on the basis of PASS prediction. PASS prediction (Table 2) of the rhamnopyranoside derivatives **4**–**8** were 0.48 < *P*_a_ < 0.60 in antibacterial and 0.65 < *P*_a_ < 0.73 in antifungal, where rhamnopyranoside derivatives **4**–**8** were more potent against phytopathogenic fungi as compared to bacterial pathogens.

### 2.5. Antimicrobial Studies

The rhamnopyranoside derivatives **4**–**8** were employed as test chemicals for in vitro antimicrobial studies. In Table 3 we presented the in vitro zone of inhibition against four Gram-positive and four Gram-negative bacteria compounds **4**–**8**. These results, as summarized in Table 3, indicated that the rhamnopyranoside derivatives **4**–**8** were less active against both the Gram-positive and Gram-negative pathogens although 4-*O*-benzoate **7**, having more aromatic moiety, was found more active and exhibited considerable inhibition against Gram-negative bacterial pathogens *Escherichia coli* (8 mm) and *Klebsiella pneumoniae* (9 mm).

The results of the percentage inhibitions of mycelial growth of two tested fungi (one plant pathogenic and one human pathogenic) due to the effect of the test chemicals **4**–**8** are summarized in Table 4. It was found that, among the test chemicals, chemicals **6** (63.8%) and **7** (65%) showed relatively higher inhibition than the standard antibiotic, Nystatin (63.1%). An important observation was that the rhamnopyranosides were found comparatively more prone against the tested fungal pathogens than that of bacterial organisms. This result is significant agreement with PASS prediction (Table 2) that rhamnopyranoside derivatives **4**–**8** were 0.48 < *P*_a_ < 0.60 in antibacterial and 0.65 < *P*_a_ < 0.73 in antifungal, suggesting rhamnopyranoside derivatives **4**–**8** were more prone towards fungal inhibition as compared to bacterial inhibition.

### 2.6. Structure Activity Relationship (SAR)

It is necessary to understand the mechanisms of antimicrobial action for the design and improved antimicrobial agents. Considering the above fact, we attempted to derive SAR of the rhamnopyranosides on the basis of our PASS and in vitro experimental results. It was evident from Table 2, Table 3 and Table 4 that incorporation of benzoyl group, especially in the C-4 position, as in **6** and **7**, increased the antimicrobial potentiality of benzyl α-l-rhamnopyranoside **4**. In addition, the rhamnopyranoside derivatives **4**–**8** were more prone towards fungal inhibition as compared to bacterial inhibition.

According to Hunt [40], the potency of alcoholic compounds against organisms is directly related to their lipid solubility property. Especially, the hydrophobic interaction between alkyl chains from alcoholic compounds and lipid regions in the organisms’ membrane. As this hydrophobic interaction is directly related to membrane permeation [40,41], the hydrophobicity of chemicals is considered a prominent parameter which affects bioactivity, toxicity or alteration of membrane integrity causing death of the organisms. Thus, it was expected that partially or fully acylated compounds **6** and **8,** with more hydrophobic nature, might show better toxicity against the tested microorganisms as compared to that of **4**, **5** and **7** having more hydroxyl groups. Although hydrophobic compound **6** exhibited antimicrobial activities accordingly, to our surprise, the other hydrophobic compound **8** didn’t show any activity against both types of tested organisms. On the other hand, hydrophilic compound **7** showed better activities. This is probably due to steric hindrance of compound **8** with two acetyl groups and one bulky benzoyl group.

## 3. Materials and Methods

L-Rhamnose and all reagents used were commercially available (Sigma-Aldrich and Merck) and were used as received unless otherwise specified. Melting points (mp) of the solid samples were determined on an electrothermal melting point apparatus and are uncorrected. All evaporations were carried out under reduced pressure on a Büchi rotary evaporator (R 100, Buchi Labortechnik AG, Flawil, Switzerland). Thin layer chromatography (TLC) was carried out on Kieselgel GF_254_ coated on glass plate. Visualization of TLC was accomplished by spraying with 1% H_2_SO_4_ and subsequent heating the plates at high temperature (150–200 °C) till colour spot of compound(s) appeared. Chromatographic purification was performed on a long column with silica gel (100–200 mesh). FT-IR spectral data were obtained from the Department of Chemistry, University of Chittagong and recorded on a FT-IR spectrophotometer (IR Prestige-21, Shimadzu, Kyoto, Japan) using CHCl_3_ and KBr techniques. For ^1^H (400 MHz) NMR spectra we used CD_3_OD or CDCl_3_ as a solvent and recorded at Bangladesh Council for Scientific and Industrial Research (BCSIR) Laboratories, Dhaka, Bangladesh. Chemical shifts were reported in δ unit (ppm) with reference to tetramethylsilane (TMS) as an internal standard and *J* values are given in Hz. The Supplementary Data section reports the IR and ^1^H NMR spectral data (Figures S1–S13) of compounds **4**–**8**.

### 3.1. General Procedure: Synthesis

*Synthesis of benzyl α-L-rhamnopyranoside* (**4**): (a) Direct method: L-rrhamnose and anhydrous benzyl alcohol with Amberlite IR 120 (H^+^) resin were stirred at 120 °C for 30 h to furnish the title compound **4** in 82% yield as a thick syrup using literature procedure [20]. *R*_f_ = 0.52 (CHCl_3_/MeOH = 10/1); FT-IR (CHCl_3_): 3480–3310 cm^−1^ (br, OH); ^1^H NMR (400 MHz, CD_3_OD): δ 7.12–7.28 (5H, m, Ar-*H*), 4.75 (1H, s, H-1), 4.68 (1H, d, *J* = 12.0 Hz, PhC*H*_A_H_B_), 4.59 (1H, m, H-2), 4.50 (1H, d, *J* = 12.0 Hz, PhCH_A_*H*_B_), 3.76–3.84 (1H, m, H-3), 3.57–3.69 (1H, m, H-5), 3.38 (1H, t, *J* = 10.6 Hz, H-4), 3.27–3.32 (3H, br s, exchange with D_2_O, 3 × O*H*), 1.26 (3H, d, *J* = 6.4 Hz, 6-C*H*_3_). The Supplementary Data section reports the FT-IR (Figure S1) and ^1^H NMR (Figures S2 and S3) spectral data of compound **4**.

(b) Microwave assisted method: L-rhamnose (finely powdered, 0.8 g, 4.873 mM) was taken in a porcelain dish followed by addition of dry benzyl alcohol (1.0 mL) and Amberlite IR 120 (H^+^) ion exchange resin (0.8 g). The mixture was mixed well with a spatula and covered with a glass plate. The reaction mixture was then placed in a domestic microwave oven and irradiated at 160 watts for 1.5 min (30 s × 3). Progress of the reaction was monitored every 30 s intervals by TLC (CHCl_3_/MeOH = 10/1). The reaction mixture was filtered and the filtrate was evaporated under reduced pressure to leave a thick syrup. The syrup was then purified by a short silica gel column to give pure benzyl rhamnopyranoside (1.19 g, 96%) as brownish thick liquid. The IR and ^1^H NMR spectra of this compound were similar to that of earlier prepared **4** by conventional method (direct method).

*Synthesis of benzyl 2,3-O-isopropylidene-α-L-rhamnopyranoside* (**5**): To a round bottom flask, benzyl α-L-rhamnopyranoside **4** (2.0 g, 7.865 mM) and excess 2,2-dimethoxyprpane (DMP, 40 mL) was stirred well and added catalytic amount of *p*-toluenesulfonic acid (*p*-TSA, 0.02 mg). Here, DMP acts both as a solvent and as a reagent. The reaction mixture was refluxed for 30 min. The mixture was then cooled, 10% NaHCO_3_ solution (2 mL) added and extracted with ethyl acetate (3 × 5 mL). The ethyl acetate layer was dried (MgSO_4_) and concentrated in vacuum to leave a thick syrup which on chromatographic purification elution with *n*-hexane/ethyl acetate = 10/1 gave compound **5** as a clear oil (1.829 g, 79%). *R*_f_ = 0.45 (*n*-hexane/ethyl acetate = 4/1); FT-IR (CHCl_3_): 3450–3300 (br, OH), 1381 cm^−1^ [C(CH_3_)_2_]; ^1^H NMR (400 MHz, CDCl_3_): δ 7.09–7.36 (5H, m, Ar-*H*), 4.92 (1H, s, H-1), 4.72 (1H, d, *J* = 11.8 Hz, PhC*H*_A_H_B_), 4.70 (1H, d, *J* = 5.0 Hz, H-2), 4.66 (1H, dd [apparent t], *J* = 6.9 and 5.8 Hz, H-3), 4.58 (1H, d, *J* = 11.8 Hz, PhCH_A_*H*_B_), 4.51–4.57 (1H, m, H-5), 4.42–4.48 (1H, m, H-4), 1.90–2.20 (1H, br s, exchange with D_2_O, O*H*), 1.33 [3H, s, C(C*H*_3_)_2_], 1.32 [3H, s, C(C*H*_3_)_2_], 1.28 (3H, d, *J* = 6.1 Hz, 6-C*H*_3_). The Supplementary Data section reports the FT-IR (Figure S4) and ^1^H NMR (Figure S5) spectral data of compound **5**.

### 3.2. General Procedure for Acylation

At first, benzyl rhamnopyranoside, having hydroxyl group(s) was dissolved in dry pyridine (~1 mL). The solution was cooled to 0 °C and acyl halide added drop wise with continuous stirring. The reaction mixture was allowed to attain room temperature and 4-dimethylaminopyridine (DMAP, catalytic) added. The mixture was further stirred for 10–16 h and TLC indicated the formation of a faster moving product. Excess acyl halide (reagent) was decomposed by addition of little amount of ice water (~1 mL). The organic product was extracted with dichloromethane (DCM, 3 × 4 mL) and this organic layer was washed successively with 5% hydrochloric acid, saturated aqueous sodium hydrogen carbonate solution and brine. The organic layer was dried over magnesium sulphate and concentrated in vacuum to leave a residue. Chromatographic purification of the residue (elution with *n*-hexane/ethyl acetate) furnished the corresponding acyl ester product in the pure form.

*Synthesis of benzyl 4-O-benzoyl-2,3-O-isopropylidene-α-L-rhamnopyranoside* (**6**): White solid, mp 115–117 °C; yield 87%; *R*_f_ = 0.61 (*n*-hexane/ethyl acetate = 5/1); FT-IR (CHCl_3_): 1681 (CO), 1375 cm^−1^ [C(CH_3_)_2_]; ^1^H NMR (400 MHz, CDCl_3_): δ 8.04–8.226 (4H, m, Ar-*H*), 7.58–7.73 (2H, m, Ar-*H*), 7.35–7.52 (4H, m, Ar-*H*), 5.38 (1H, s, H-1), 5.18 (1H, dd, *J* = 10.1 and 6.9 Hz, H-4), 4.75 (1H, d, *J* = 11.9 Hz, PhC*H*_A_H_B_), 4.60 (1H, d, *J* = 11.9 Hz, PhCH_A_*H*_B_), 4.38 (1H, dd, *J* = 10.1 and 2.9 Hz, H-3), 4.28 (1H, d, *J* = 2.9 Hz, H-2), 3.88–4.05 (1H, m, H-5), 1.63 [3H, s, C(C*H*_3_)_2_], 1.37 [3H, s, C(C*H*_3_)_2_], 1.25 (3H, d, *J* = 6.2 Hz, 6-C*H*_3_). The Supplementary Data section reports the FT-IR (Figure S6) and ^1^H NMR (Figure S7) spectral data of compound **6**.

*Synthesis of benzyl 4-O-benzoyl-α-L-rhamnopyranoside* (**7**): A solution of 4-*O*-benzoate **6** (1.8 g, 4.518 mM) in glacial acetic acid (20 mL) was stirred at 40 °C for 12 h. TLC examination indicated the formation of a slower moving product having *R*_f_ = 0.47 (*n*-hexane/ethyl acetate = 2/1). The reaction mixture was subjected for vacuum evaporation on rotary evaporator to remove acetic acid. Traces of acetic acid were also removed by co-evaporation with toluene (3 × 3 mL) to leave a thick residue which on chromatography with *n*-hexane/ethyl acetate (5/1) furnished diol **7** (1.20 g, 74%) as a colorless semi-solid. Our effort for crystallization of the semi-solid was unsuccessful. *R*_f_ = 0.47 (*n*-hexane/ethyl acetate = 2/1); FT-IR (CHCl_3_): 3600–3300 (br, OH), 1630 cm^−1^ (CO); ^1^H NMR (400 MHz, CDCl_3_): δ 8.05 (2H, d, *J* = 7.6 Hz, Ar-*H*), 7.60 (1H, t, *J* = 7.4 Hz, Ar-*H*), 7.44 (2H, t, *J* = 7.2 Hz, Ar-*H*), 7.28–7.39 (5H, m, Ar-*H*), 5.06 (1H, t, *J* = 10.0 Hz, H-4), 4.97 (1H, s, H-1), 4.76 (1H, d, *J* = 11.8 Hz, PhC*H*_A_H_B_), 4.57 (1H, d, *J* = 11.8 Hz, PhCH_A_*H*_B_), 4.09 (1H, d, *J* = 3.4 Hz, H-2), 4.07 (1H, dd, *J* = 9.6 and 3.4 Hz, H-3), 3.96–4.05 (1H, m, H-5), 1.67–2.19 (2H, br s, exchange with D_2_O, 2 × O*H*), 1.29 (3H, d, *J* = 6.0 Hz, 6-C*H*_3_). The Supplementary Data section reports the FT-IR (Figure S8) and ^1^H NMR (Figures S9 and S10) spectral data of compound **7**.

*Synthesis of benzyl 2,3-di-O-acetyl-4-O-benzoyl-α-L-rhamnopyranoside* (**8**): Thick syrup; yield 92%; *R_f_* = 0.57 (*n*-hexane/ethyl acetate = 5/1); FT-IR (KBr): 1751, 1732, 1716 cm^−1^ (CO); ^1^H NMR (400 MHz, CDCl_3_): δ 8.00 (2H, d, *J* = 8.0 Hz, Ar-*H*), 7.57 (1H, t, *J* = 7.5 Hz, Ar-*H*), 7.44 (2H, t, *J* = 7.2 Hz, Ar-*H*), 7.31–7.39 (5H, m, Ar-*H*), 5.54 (1H, dd, *J* = 10.0 and 3.2 Hz, H-3), 5.36 (1H, t, *J* = 10.0 Hz, H-4), 5.33 (1H, d, *J* = 3.2 Hz, H-2), 4.86 (1H, s, H-1), 4.75 (1H, d, *J* = 12.0 Hz, PhC*H*_A_H_B_), 4.59 (1H, d, *J* = 12.0 Hz, PhCH_A_*H*_B_), 4.05–4.13 (1H, m, H-5), 2.16 (3H, s, COC*H*_3_), 1.88 (3H, s, COC*H*_3_), 1.26 (3H, d, *J* = 6.4 Hz, 6-C*H*_3_). The Supplementary Data section reports the FT-IR (Figure S11) and ^1^H NMR (Figures S12 and S13) spectral data of compound **8**.

### 3.3. Test Human and Phytopathogens

The newly synthesized rhamnopyranoside derivatives **4**–**8** were tested against eight human pathogenic bacteria (four Gram-positive and four Gram-negative). Gram-positive organisms were *Bacillus cereus* BTCC 19, *Bacillus megaterium* BTCC 18, *Bacillus subtilis* BTCC 17 and *Staphylococcus aureus* ATCC 6538. Gram-negative pathogens were *Escherichia coli* ATCC 25922, *Klebsiella pneumoniae*, *Pseudomonas aeruginosa* *CRL* (ICDDR,B) and *Salmonella typhi* AE 14612. For in vitro mycelial growth test, two plant pathogenic fungi were selected viz. *Aspergillus brasiliensis* ATCC 16404 and *Candida albicans* ATCC.

### 3.4. Antimicrobial Screening Procedure

Screening of antibacterial activity: The disc diffusion method [8] was followed for the detection of antibacterial activities. For the culture of bacterial organisms Muller-Hinton medium (agar and broth) was prepared and used. The petri dishes (plates) were incubated at 37 °C for two days. We used dimethylformamide (DMF) (Sigma-Aldrich, Taufkirchen, Germany) as a solvent. Hence, 2% solution of each test chemicals in DMF were prepared and used for antibacterial evaluation. For accuracy, we maintained proper control only with DMF without chemicals. For activity test, 500 μL bacterial culture was used. Each experiment against each organism was conducted three times and the average value was shown in the Table 3 and Table 4. The standard antibiotic ampicillin (50 μg/disc, β-lactam antibiotic used for bacterial infections, Brand name Ficillin, Sanofi-Aventis, Dhaka, Bangladesh), was used as a positive control and compared with tested chemicals under identical conditions.

Screening of mycelial growth: The in vitro antifungal activities of the rhamnopyranosides **4**–**8** were investigated according to food poisoning technique [10,11]. For fungal culture, we used sabouraud (agar and broth, PDA) medium. For activity test, 5 mm diameter fungal mate were inoculated in the plate. Linear mycelial growth of fungus was measured after 3–5 days of incubation. The percentage inhibition of radial mycelial growth of the test fungus was calculated using the following equation:

$$I=\;\left\{\frac{C-T}{C}\right\}\;\times 100$$

where *I*, percentage of inhibition, *C* = diameter of the fungal colony in control (DMF), and *T* = diameter of the fungal colony in treatment. The results were compared with the standard antifungal antibiotic nystatin (100 μg/mL medium, brand name Candex, Square Pharmaceuticals Ltd., Dhaka, Bangladesh).

## 4. Conclusions

Thus, selective benzoylation of benzyl α-l-rhamnopyranoside **4** at C-4 position was conducted successfully using protection deprotection technique. Initially, **4** was prepared using conventional glycosidation technique as well as microwave irradiation method. Benzyl rhamnopyranoside **4** on acetonide protection followed by 4-*O*-benzylation and deacetonation gave the desired benzyl 4-*O*-benzoyl-α-l-rhamnopyranoside **7**. For structural elucidation and to get newer derivatives of **7**, we prepared 2,3-di-*O*-acetate **8**. Conformational study revealed that acetonide protected rhamnopyranoside **5** and **6** showed slight distortion from the regular ^1^C_4_ conformational structure. In vitro antimicrobial study showed that the rhamnopyranosides were more prone against pathogenic fungi as compared to pathogenic bacteria. SAR study concluded that incorporation of benzoyl group at C-4 position of rhamnopyranose framework increased the antimicrobial potentiality of the benzyl α-l-rhamnopyranoside **4**. PASS prediction of the rhamnopyranoside derivatives **4**–**8** were *P*_a_ < 0.6 in antibacterial and *P*_a_ < 0.73 in antifungal, which are confirmed experimentally where rhamnopyranoside derivatives **4**–**8** were more effective against fungal organisms as compared to pathogenic bacterial thus, there is a more than 50% chance that the rhamnopyranoside derivative structures have not been reported with this activity, making it a possible valuable lead compound.

## Figures and Tables

**Table 1:** Coupling constants of rhamnopyranosides **5**–**8**.

Compounds	Coupling Constants (Hz)		
	*J*_2,3_	*J*_3,4_	*J*_4,5_
5	5.8	6.9	–
6	2.9	10.1	6.9
7	3.4	9.6	10.0
8	3.2	10.0	10.0

–: unable to calculate *J* value from the spectrum.

**Table 2:** Predicted biological activity of synthesized compounds using prediction of activity spectra for substances (PASS) software.

Compound No.	Biological Activity			
	Antibacterial		Antifungal	
	*P*_a_	*P*_i_	*P*_a_	*P*_i_
**4**	0.561	0.011	0.654	0.013
**5**	0.495	0.017	0.733	0.008
**6**	0.479	0.018	0.715	0.009
**7**	0.582	0.010	0.665	0.012
**8**	0.608	0.008	0.670	0.012

*P*_a_, probability ‘to be active’; *P*_i_, probability ‘to be inactive’.

**Table 3:** Inhibition against bacterial organism by the rhamnopyranosides **4**–**8**.

Name of Bacteria	Diameter of Zone of Inhibition in mm, 50 μg/dw/disc					
	4	5	6	7	8	Ampicillin **
*Bacillus cereus*	NI	08	NI	NI	06	22 *
*Bacillus megaterium*	NI	NI	10	NI	NI	19
*Bacillus subtilis*	NI	NI	11	NI	08	25 *
*Staphylococcus aureus*	NI	NI	NI	NI	NI	21 *
*Escherichia coli*	NI	NI	NI	08	NI	25 *
*Klebsiella pneumoniae*	NI	06	NI	09	NI	22P
*Pseudomonas aeruginosa*	NI	NI	NI	NI	NI	17
*Salmonella typhi*	NI	NI	NI	NI	NI	13 *

* good inhibition “NI indicates no inhibition” ** indicates “standard antibiotic” dw means dry weight. Each value is the average of three replicates.

**Table 4:** Antifungal activities due to the rhamnopyranoside derivatives **4**–**8**.

Name of Fungus	% Inhibition of Fungal Mycelial Growth, Sample 100 μg/dw/mL PDA					
	4	5	6	7	8	Nystatin **
*Aspergillus brasiliensis*	35.0	30.0	25.0	20.0	NI	66.4 *
*Candida albicans*	60.0 *	58.8	63.8 *	65.0 *	NI	63.1 *

* good inhibition “NI indicates no inhibition” ** indicates standard “antibiotic” dw means dry weight; PDA means potato dextrose agar. Each value is the average of three replicates.

## Supplementary Materials

Supplementary materials can be found at www.mdpi.com/1422-0067/17/9/1412/s1.
