# Supplementary material for: Synthesis, PASS-Predication and in Vitro Antimicrobial Activity of Benzyl 4-O-benzoyl-α-l-rhamnopyranoside Derivatives
Source: Int J Mol Sci. 2016 Aug 27;17(9):1412. doi: 10.3390/ijms17091412 (PMC5037692; doi:10.3390/ijms17091412)
Supplement: Supplementary file 1 [file ijms-17-01412-s001.pdf]

Supplementary Materials: Synthesis, PASS-Predication and in Vitro Antimicrobial Activity of Benzyl 4-*O*-benzoyl-α-L-rhamnopyranoside Derivatives

Mohammed Mahbubul Matin, Amit R. Nath, Omar Saad, Mohammad M. H. Bhuiyan, Farkaad A. Kadir, Sharifah Bee Abd Hamid, Abeer A. Alhadi, Md. Eaqub Ali and Wageeh A. Yehye

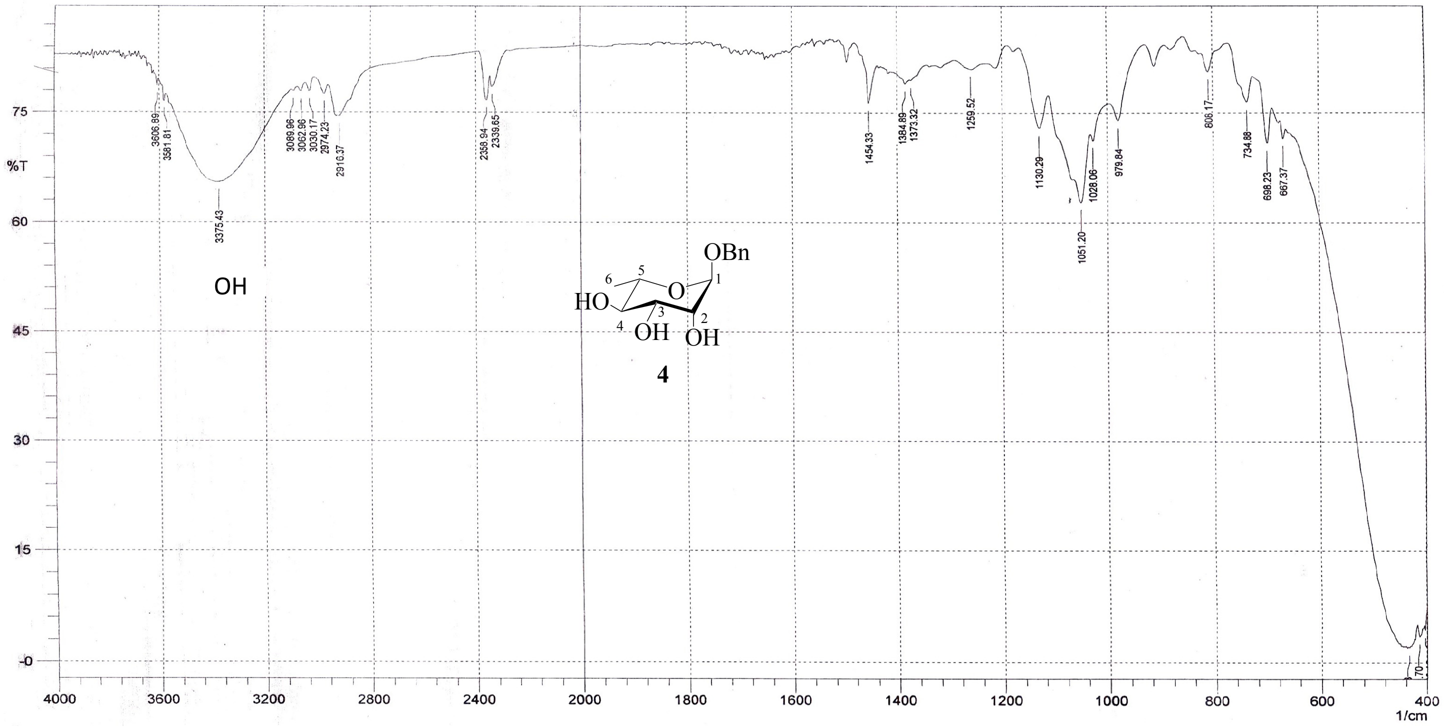

Figure S1. FT-IR ($CHCl_3$) spectrum of the rhamnopyranoside (**4**).

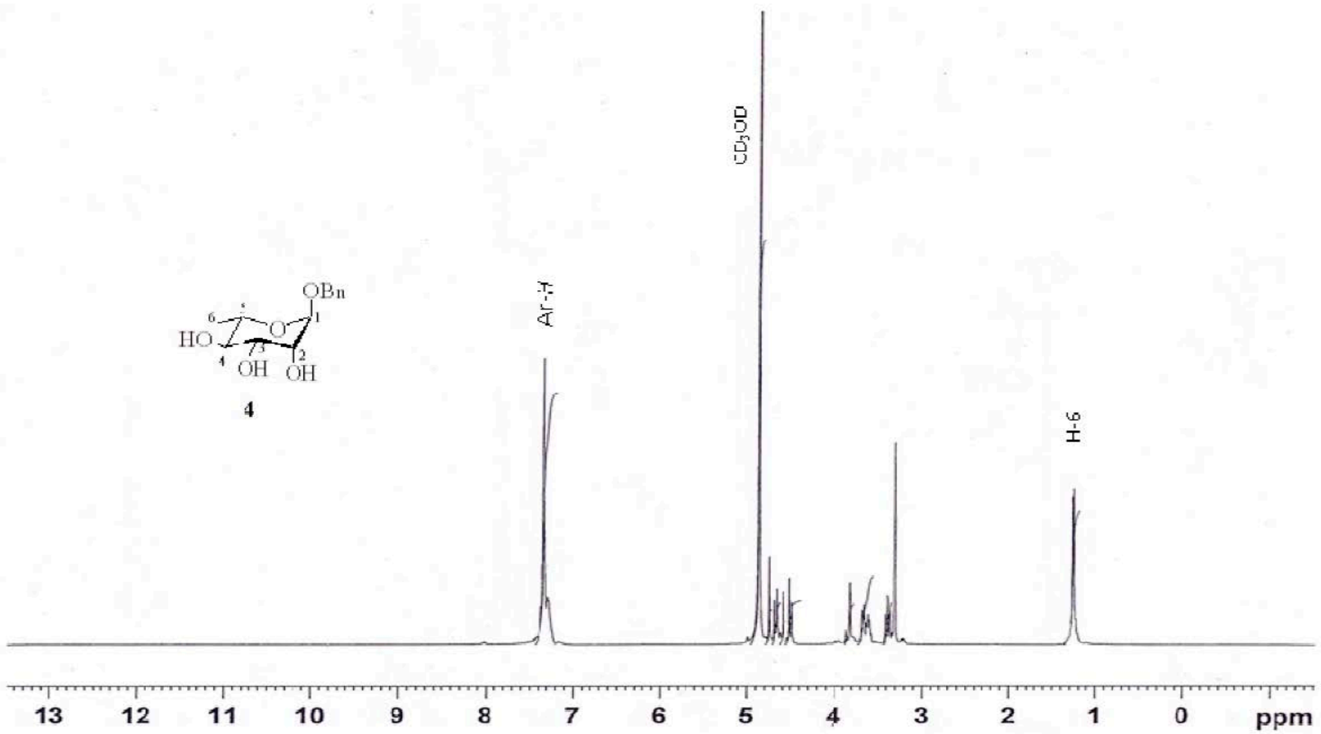

Figure S2. ^{1}H NMR (400 MHz, CD_3OD) spectrum of the rhamnopyranoside (**4**).

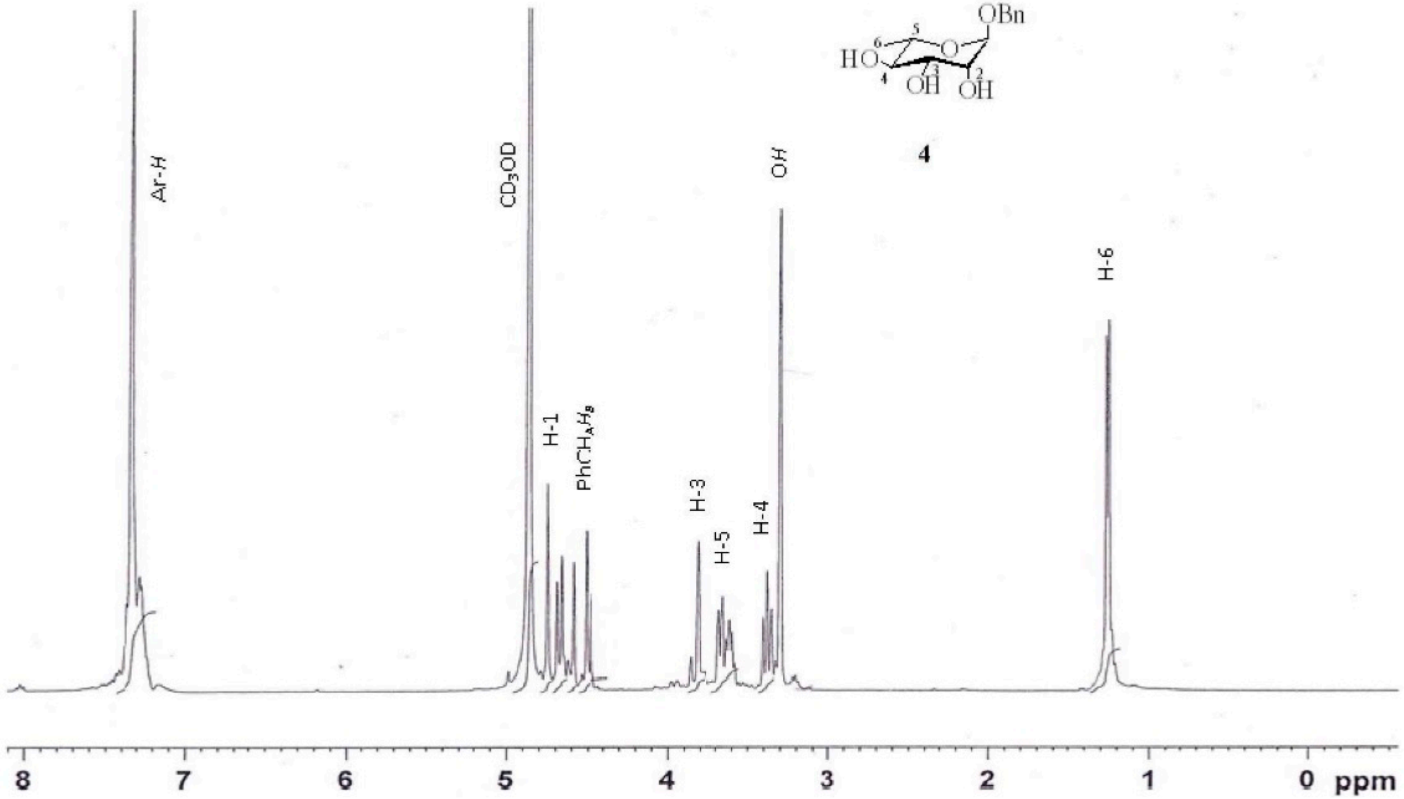

Figure S3. Expansion of ^{1}H NMR (400 MHz, CD_3OD) spectrum of the rhamnopyranoside (**4**).

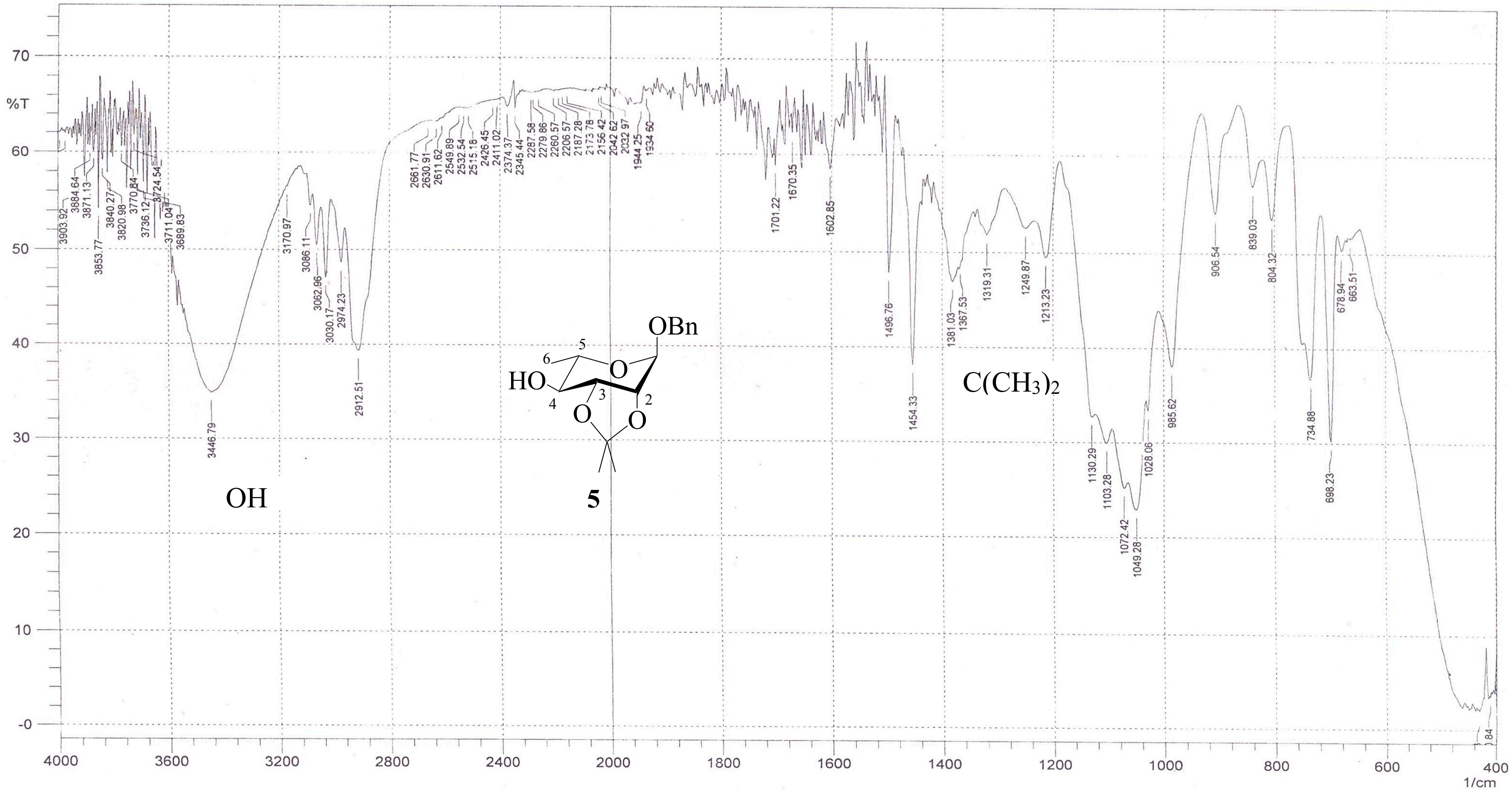

Figure S4. FT-IR ($CHCl_3$) spectrum of the compound (**5**).

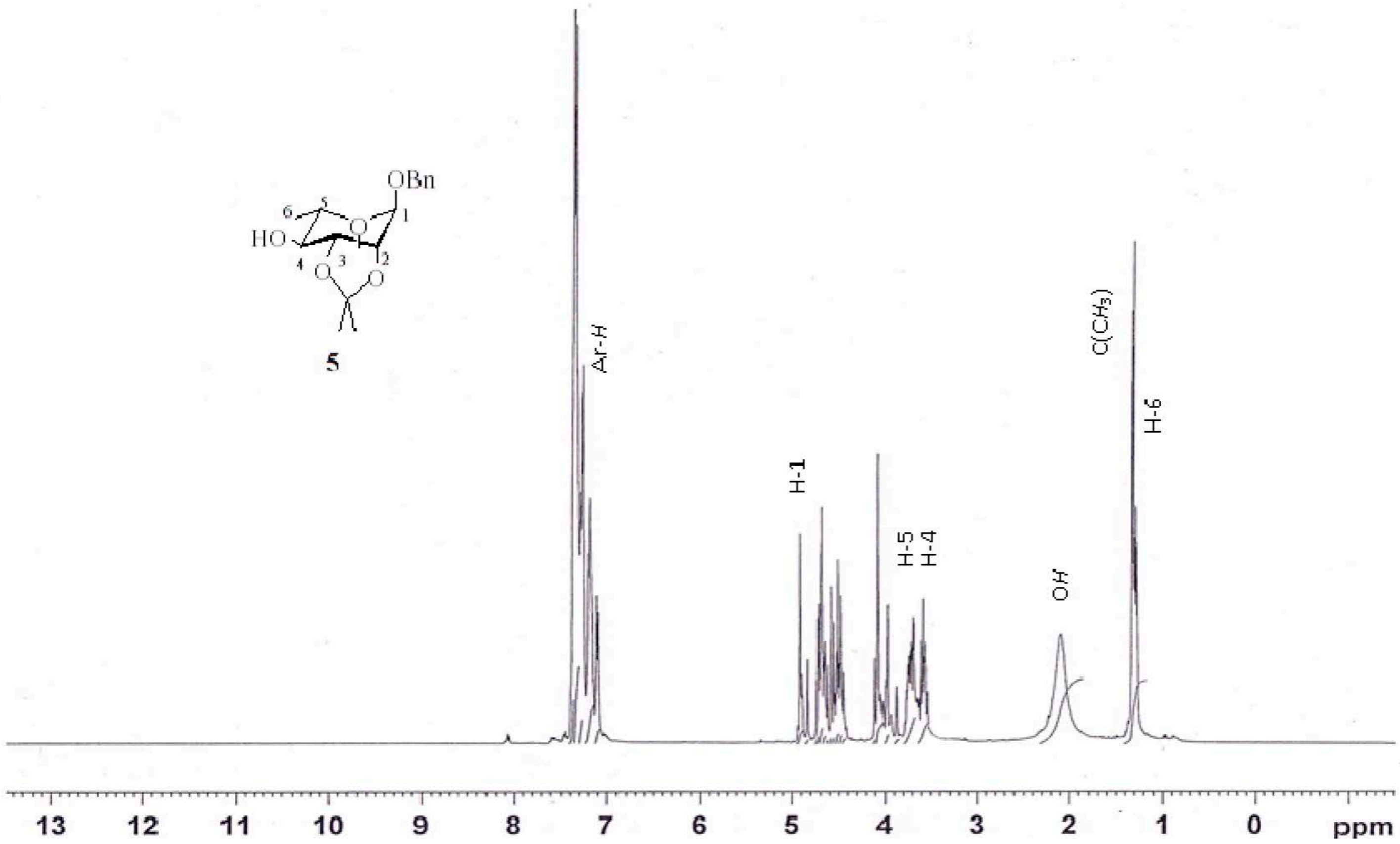

Figure S5. ^{1}H NMR (400 MHz, $CDCl_3$) spectrum of the compound (**5**).

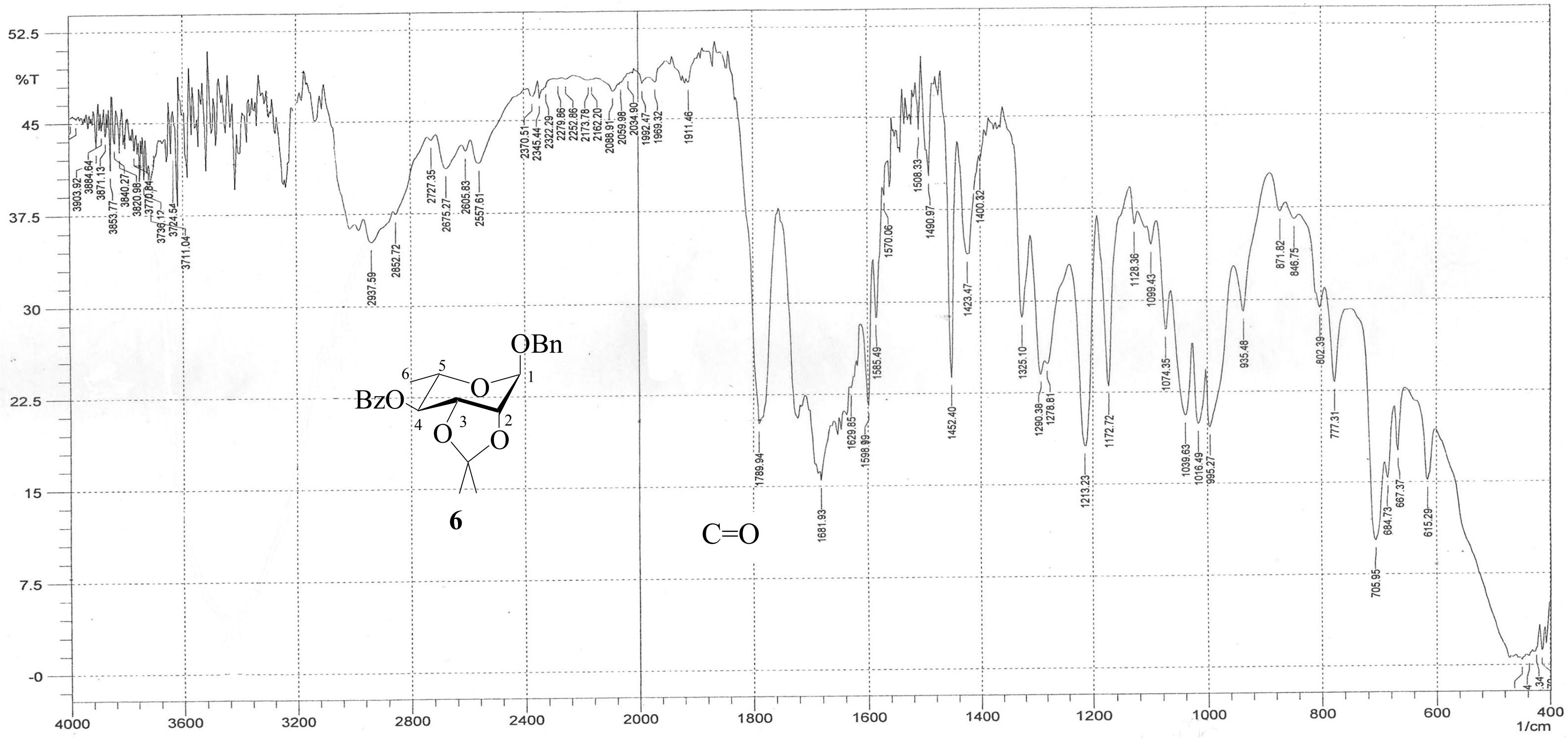

Figure S6. FT-IR ($CHCl_3$) spectrum of the compound (**6**).

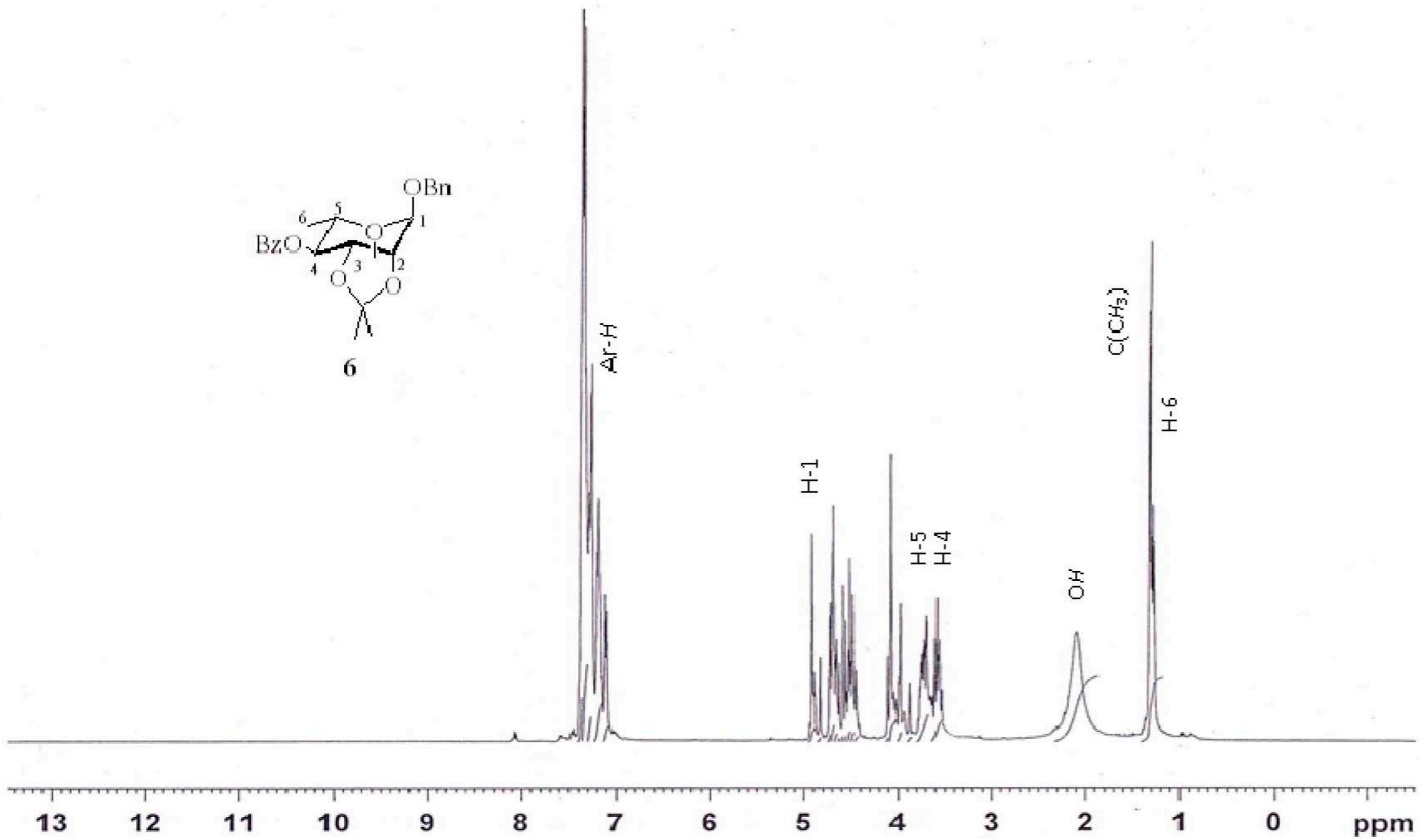

Figure S7. ^1H NMR (400 MHz, $CDCl_3$) spectrum of the compound (**6**).

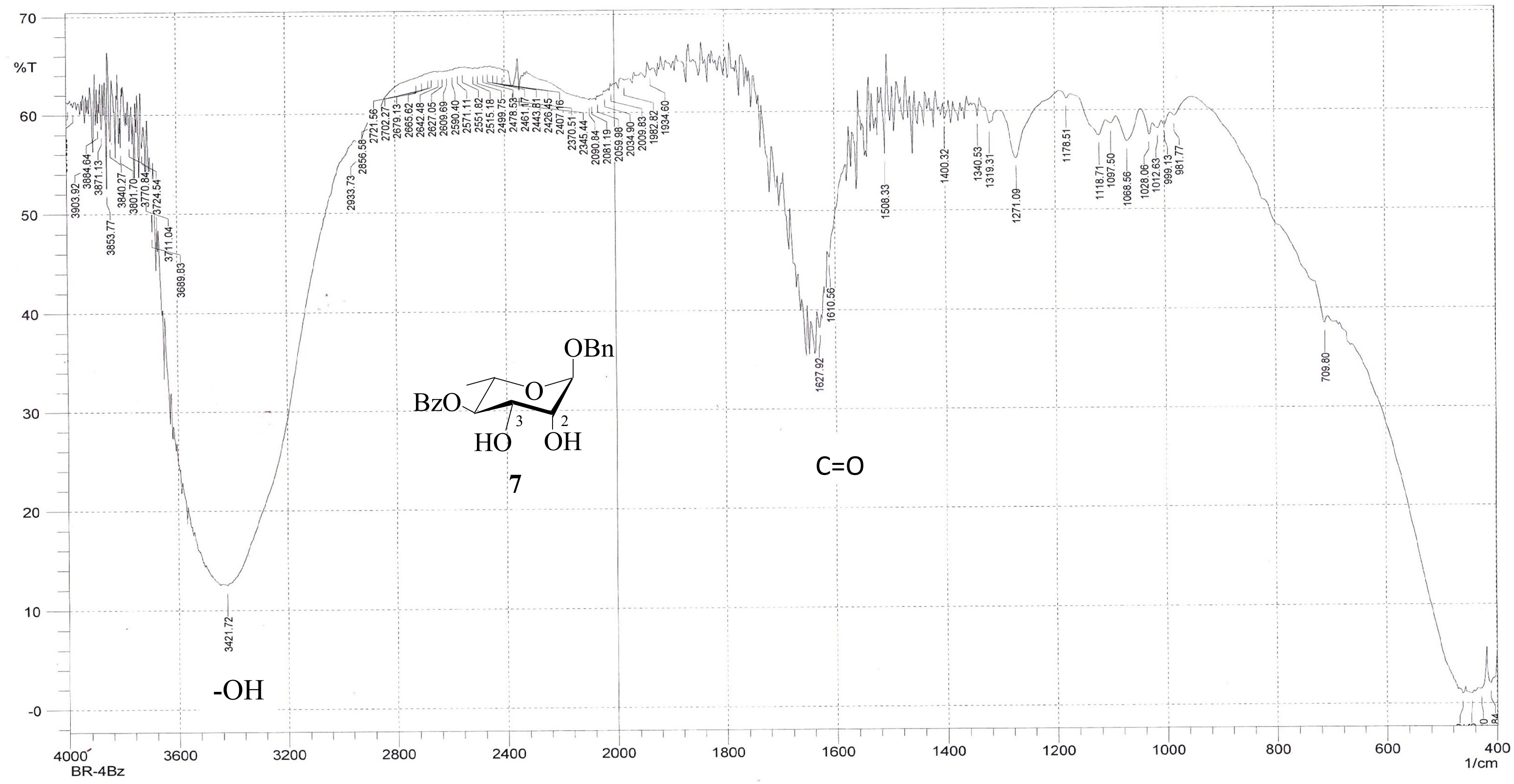

Figure S8. FT-IR ($CHCl_3$) spectrum of the compound (**7**).

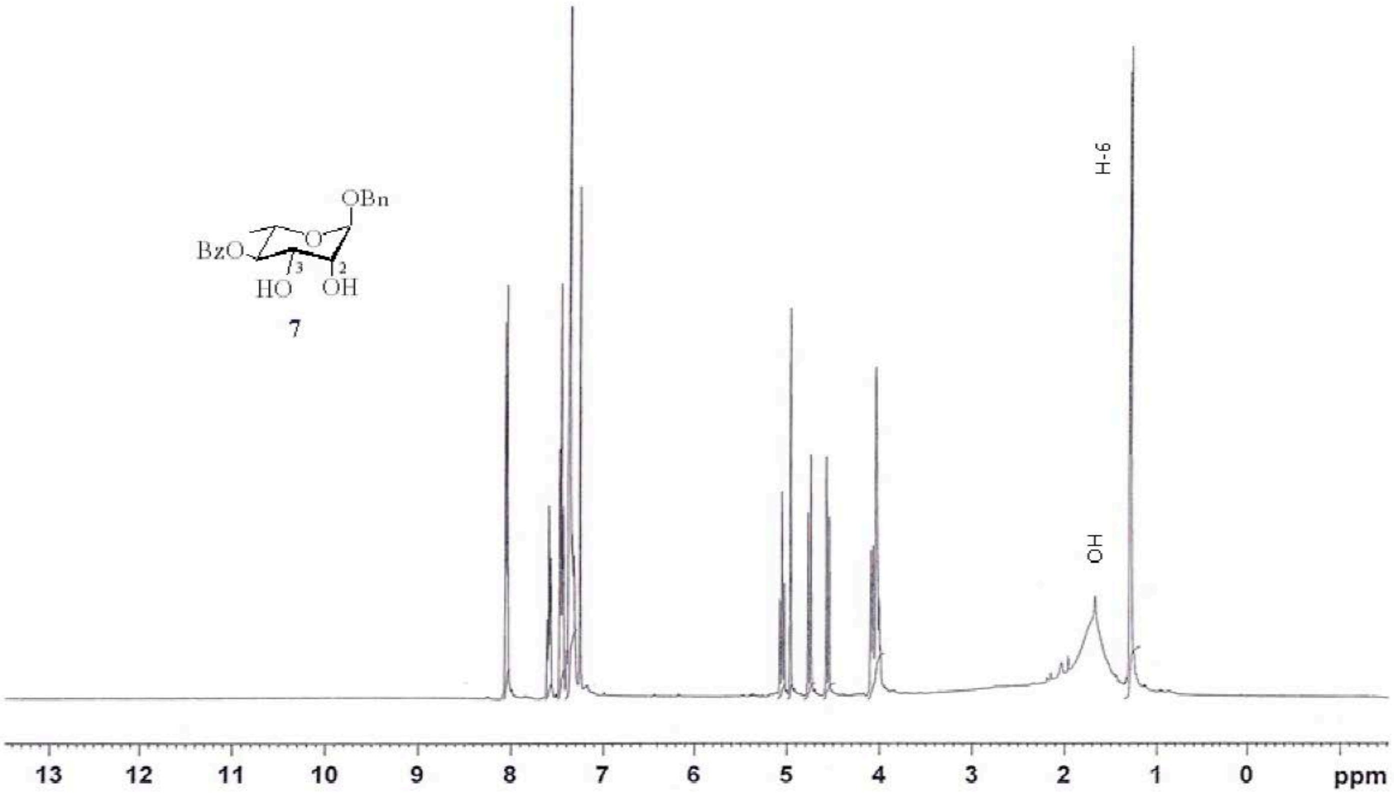

Figure S9. ^{1}H NMR (400 MHz, $CDCl_3$) spectrum of the 4-*O*-benzate (**7**).

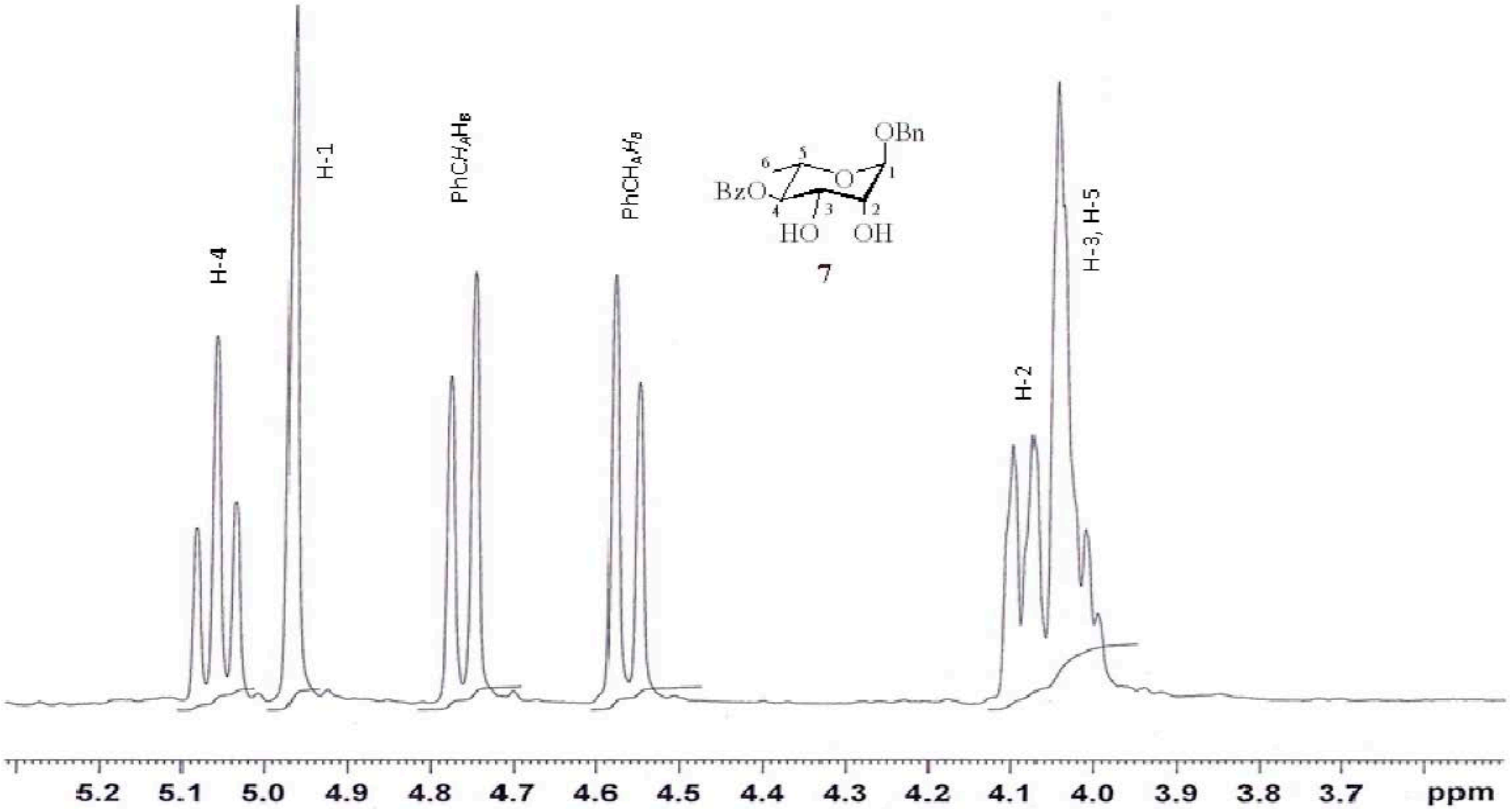

Figure S10. Expansion of ^{1}H NMR (400 MHz, $CDCl_3$) spectrum of the 4-*O*-benzate (**7**).

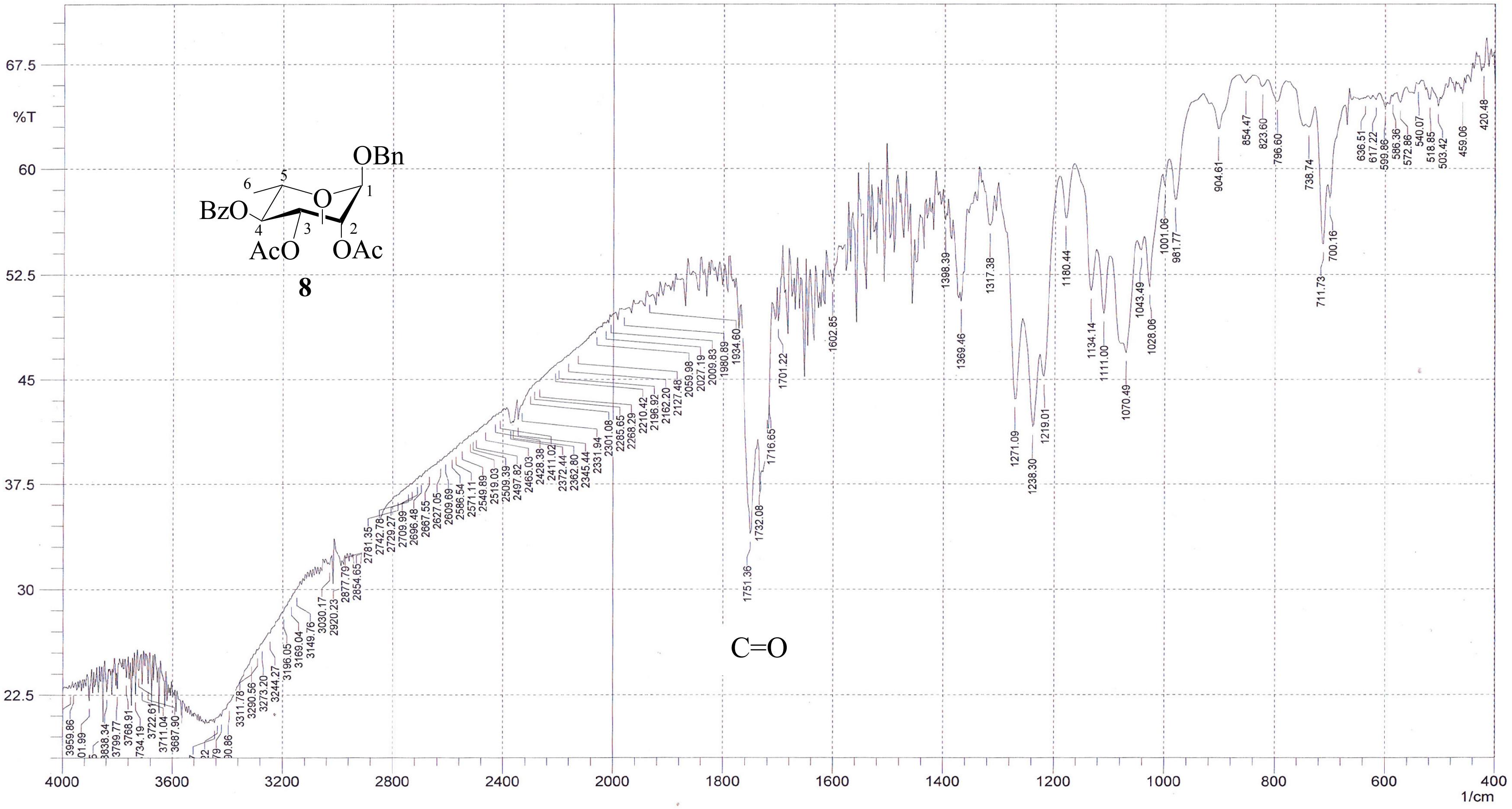

Figure S11. FT-IR (KBr) spectrum of the compound (**8**).

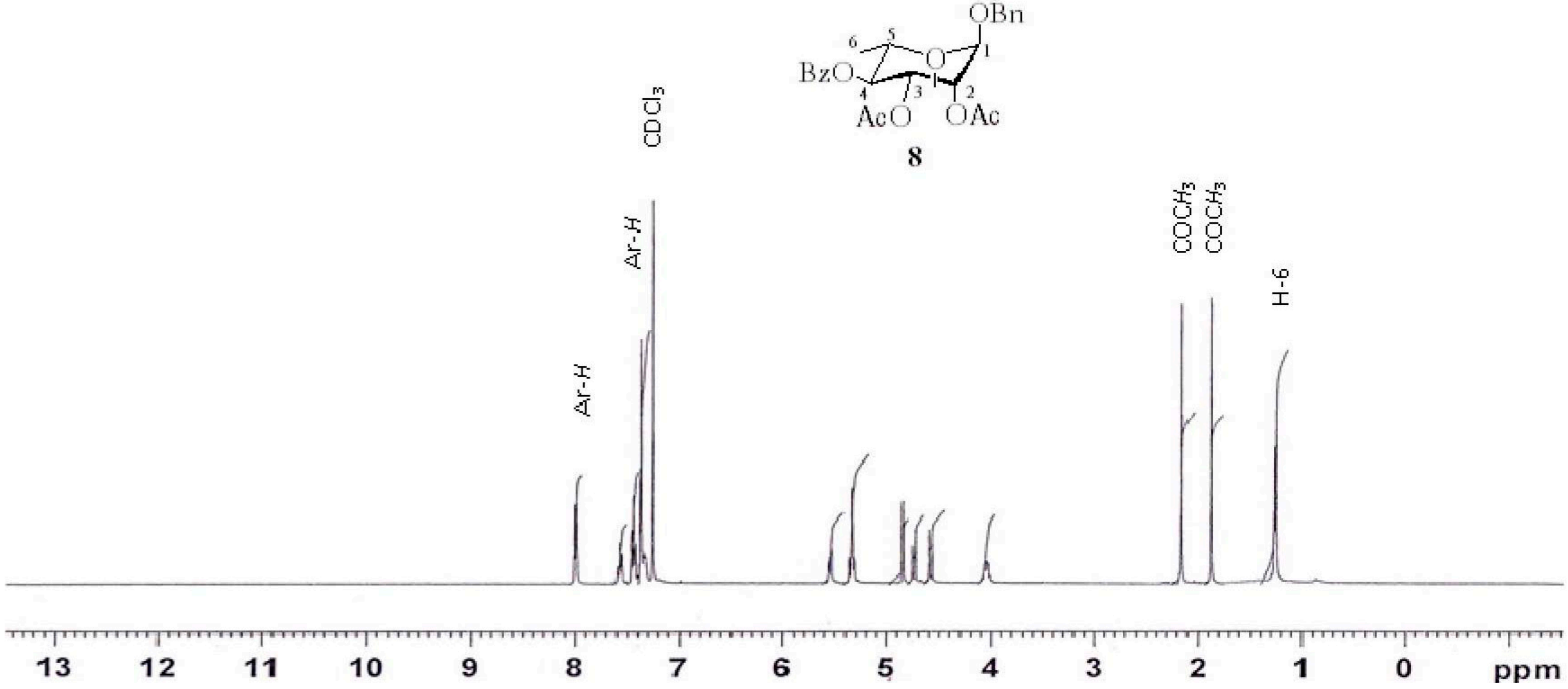

Figure S12. ^{1}H NMR (400 MHz, $CDCl_3$) spectrum of the compound (**8**).

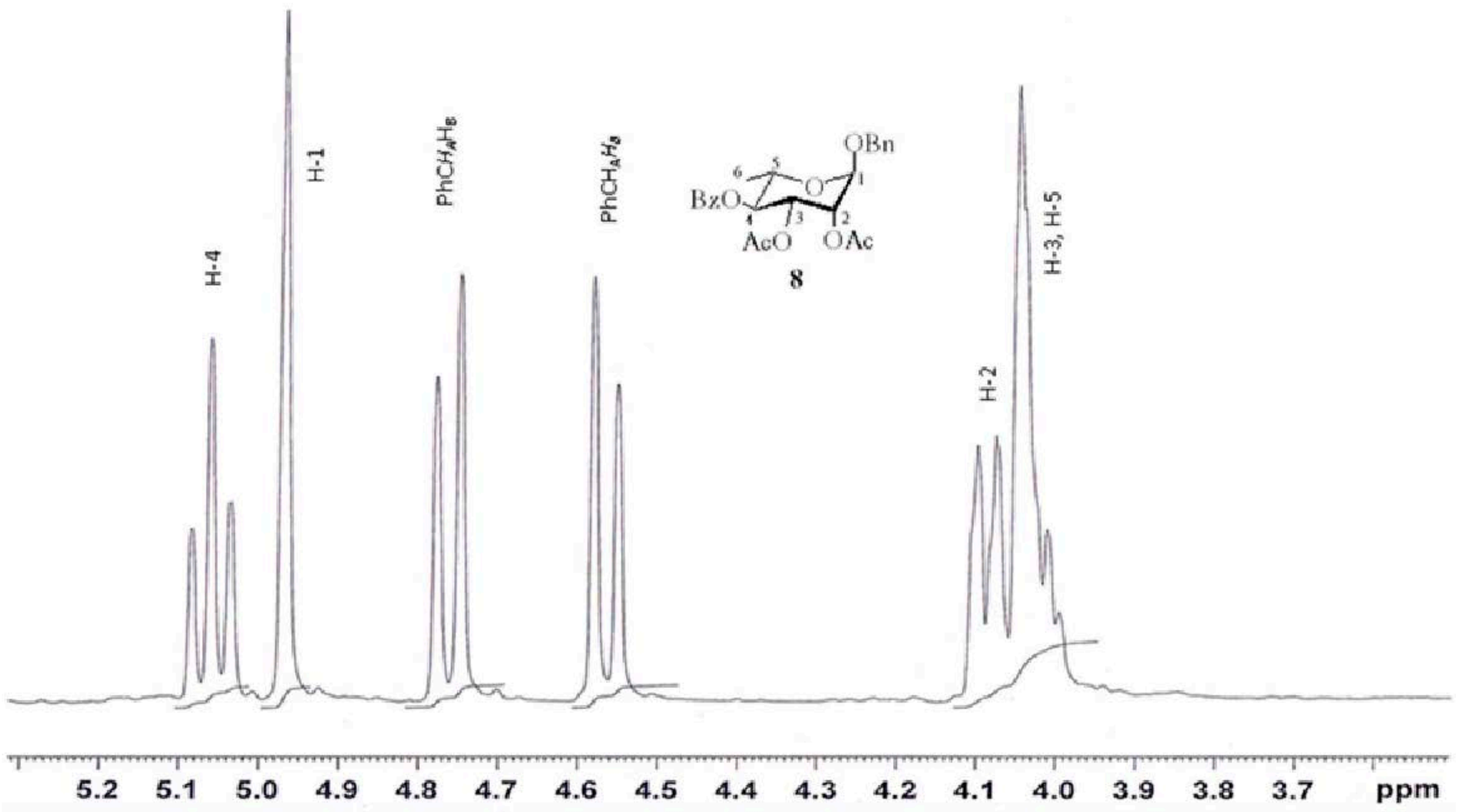

Figure S13. Expansion of ^{1}H NMR (400 MHz, $CDCl_3$) spectrum of the compound (**8**).
